# Screen-Printed Electrode Surface Modification with NiCo_2_O_4_/RGO Nanocomposite for Hydroxylamine Detection

**DOI:** 10.3390/nano11123208

**Published:** 2021-11-26

**Authors:** Somayeh Tajik, Hadi Beitollahi, Sayed ali Ahmadi, Mohammad Bagher Askari, Antonio Di Bartolomeo

**Affiliations:** 1 Research Center of Tropical and Infectious Diseases, Kerman University of Medical Sciences, Kerman P.O. Box 76169-13555, Iran; s.tajik@kmu.ac.ir; 2 Environment Department, Institute of Science and High Technology and Environmental Sciences, Graduate University of Advanced Technology, Kerman P.O. Box 76318-85356, Iran; 3 Department of Chemistry, Kerman Branch, Islamic Azad University, Kerman P.O. Box 76351-31167, Iran; saahmadi@iauk.ac.ir; 4 Department of Physics, Faculty of Science, University of Guilan, Rasht P.O. Box 41335-1914, Iran; mbaskari@phd.guilan.ac.ir; 5 Department of Physics “E.R. Caianaiello”, University of Salerno, 84084 Fisciano, Salerno, Italy

**Keywords:** hydroxylamine, NiCo_2_O_4_/RGO nanocomposite, screen-printed electrode, voltammetry

## Abstract

We developed a novel hydroxylamine sensor through the surface modification of screen-printed electrode (SPE) with NiCo_2_O_4_ nanoparticles/reduced graphene oxide (RGO) nanocomposite (NiCo_2_O_4_/RGO/SPE). We assessed the electrochemical response of hydroxylamine on the as-fabricated sensor, confirming the high electrocatalytic impact of hydroxylamine oxidation. The electrode produced sensitively responded to hydroxylamine under optimized conditions, with a low limit of detection (2.0 nM) and broad linear dynamic range (0.007–385.0 µM). The presence of NiCo_2_O_4_ combined with the modification of RGO resulted in sensitive detection and signal amplification of hydroxylamine oxidation. The proposed sensor was used to determine the existence of hydroxylamine in water samples.

## 1. Introduction

Hydroxylamine, HA or NH_2_OH, is an ammonium-containing agent that can act as an intermediate in microbial nitrogen cycle processes, generated during anaerobic ammonium oxidation and nitrification. It has also a function of reducing agent extensively present in various pharmaceutical and industrial applications [1]. Nevertheless, the presence of this agent may show moderate toxicity in humans, plants and animals with reversible and irreversible physiological side effects. The low concentrations (mM) of HA are reportedly found in a stable state for several hours at the pH value of 4.0 but for only 1 h at the pH value of 7.8 in exposure to air [2,3]. Accordingly, it is important to detect HA directly in environmental and biological specimens.

The presence of HA has been recently detected by various techniques, including gas chromatography [4], capillary electrophoresis [5], spectrophotometric [6,7] and chromatography [8,9] analysis, although they involve complex processes. Recently, due to their low cost, operational simplicity, high sensitivity, good selectivity, and fast response, electrochemical techniques have gained attention and have exhibited promising applications for HA analysis [10,11,12,13,14,15].

Screen-printed electrodes (SPEs) are disposable electrochemical electrodes with advantages like low cost, mass production, disposability and low background current, which can improve multiple shortcomings of carbon paste electrodes and glassy carbon electrodes, such as tedious cleaning procedures and memory impacts. The practical application of disposable screen-printed electrodes is limited due to shortcomings such as low reproducibility and sensitivity, but their modifiable surface is able to improve various sensing materials in terms of detection performance [16,17,18,19]. Hence, the detection of HA can be achieved using the SPEs.

Chemically modified electrodes improve mass transfer kinetics at low overpotential resulting in a decrease the interferences’ effect and avoiding surface fouling [20,21,22,23,24,25,26]. The performance of the sensors has been improved due to significant advances in nanomaterials, for example in terms of sensitivity and a wide range of detection of target molecules [27,28,29,30,31,32,33,34,35].

In the structure of the NiCo_2_O_4_ spinel, the Ni atoms occupy the tetrahedral sites, and the Co atoms occupy the octahedral sites in Co_3_O_4_. NiCo_2_O_4_, with low activation energy of electron transfer between cations, exhibits better conductivity and electrochemical features compared to pure Co_3_O_4_ and NiO. Several deficiencies have been reported for NiCo_2_O_4_, including low conductivity and easy accumulation on the electrode surface, hence resulting in poor sensitivity [36,37,38]. We can enhance the electrical conductivity and widen the surface area through the combination of composite materials. Graphene has been recently at the center of attention because of special chemical, optical and electronic properties. In particular, reduced graphene oxide (RGO) has been evaluated and applied in different fields. In electrochemistry, owing to the large surface area, high conductivity and admirable electrochemical responses, RGO has been used to load of other nanomaterials. Composites provide synergistic impacts, which are highly desired in sensor surface modification. The integration of NiCo_2_O_4_ and graphene can significantly prevent the self-accumulation of individual graphene and metal oxides. The RGO with large surface area and potent electrical conductivity forms further active sites for NiCo_2_O_4_, thereby enhancing the sensing potential of NiCo_2_O_4_ [39,40,41].

The novelty of this work concerns the observed catalytic action of the NiCo_2_O_4_/RGO nanocomposite, demonstrating the possibility to detect HA at a low potential and at higher current values. Moreover, the NiCo_2_O_4_/RGO/SPE sensor was applied to detect the HA in the different water samples.

## 2. Materials and Methods

### 2.1. Chemicals and Equipment

The electrochemical measurements were performed by a PGSTAT 302N Autolab potentiostat/galvanostat analyzer (Eco-Chemie; B.V. Kanaalweg, The Netherlands). All test conditions were monitored by General Purpose Electrochemical System (GPES) software. A three-part DropSens SPE (DRP-110, Asturias, Spain) included a graphite working electrode, a silver pseudo-reference electrode and a graphite auxiliary electrode. The solution pH values were measured by a Metrohm 710 pH meter. Double distilled water was applied to prepare all test fresh solutions. All reagents, including hydroxylamine, possessed analytical grade belonged to Merck (Darmstadt, Germany). Orthophosphoric acid and related salts were utilized to prepare all buffer solutions at the pH values (2.0 to 9.0).

### 2.2. Fabrication of NiCo_2_O_4_/RGO Nanocomposite

A modified Hummers method was used to construct the graphene oxide. To this end, the graphite powder (1 g) was first poured into sulfuric acid (200 mL) and stirred for 1 h, followed by placing in the cold-water bath and gradually adding KMnO_4_ (9 g) and stirring for 24 h. Then, the obtained mixture was added slowly with deionized water (200 mL) and subsequently with H_2_O_2_ (35 mL) to stop the oxidation. Next, the product (GO) was exposed to 0.2 M HCl and rinsed by deionized water to discard additional acid.

The NiCo_2_O_4_ nanorods were produced by dissolving cobalt acetate.4H_2_O (2 mM) and Nickel acetate.4H_2_O (1 mM) in a mixture of deionized water and ethylene glycol (in equal ratios) under the ultrasonication. Then, the mixture was gradually added with polyvinylpyrrolidone (10 mM PVP), stirred for one hour, placed in a Teflon autoclave, and dried in an oven for 16 h at 160 °C. The obtained product was washed by distilled water and ethanol, dried for 24 h at a temperature of 60 °C, and finally calcined at 400 °C for 2 h.

The NiCo_2_O_4_/RGO was constructed by dissolving GO (100 mg) into a mixture of deionized water and ethylene glycol (with equal ratio), followed by adding the PVP (10 mM) and stirring for 1 h and subsequently adding Co(Ac)_2_·4H_2_O (2 mM) and Ni(Ac)_2_·4H_2_O (1 mM) and re-stirring for 2 h. The reaction mixture was placed in the steel autoclave, dried in the oven at 160 °C for 16 h, washed with distilled water-ethanol mix, re-dried at 60 °C for 24 h, and finally calcined at 400 °C for 2 h to collect spinel NiCo_2_O_4_/RGO. The RGO:NiCo_2_O_4_ weight ratio was approximately 1:2.

### 2.3. Fabrication of Modified Electrode

A facile protocol was performed to cover the bare SPE using the NiCo_2_O_4_/RGO nanocomposite. Thus, 1 mg of NiCo_2_O_4_/RGO nanocomposite was dispersed in 1 mL aqueous solution and ultra-sonicated for half an hour to give a homogeneous solution. Then, 4 µL of prepared suspension was dropped on the surface of SPE surface. After the solvent evaporated, the electrode surface was thoroughly rinsed with deionized water to wash away the unremoved modifier and dried at room temperature. The obtained electrode was noted as NiCo_2_O_4_/RGO/SPE.

In order to investigate the available active surface area of electrodes, the cyclic voltammograms bare SPE and NiCo_2_O_4_/RGO/SPE were recorded in 1 mM K_3_Fe(CN)_6_ at different scan rates. Using the Randles–Sevcik equation [42], the electrochemical active surface area of NiCo_2_O_4_/RGO/SPE was found 0.01 cm^2^ which was about 3.2 times greater than bare SPGE.

### 2.4. Real Sample Analysis

The real specimens included river, drinking and tap water samples, which were filtered thoroughly before analysis and then different amounts of HA concentrations were added to the samples and analyzed using standard addition method.

## 3. Results

### 3.1. Determination of the NiCo_2_O_4_/RGO Nanocomposite Characteristics

**Figure 1:**
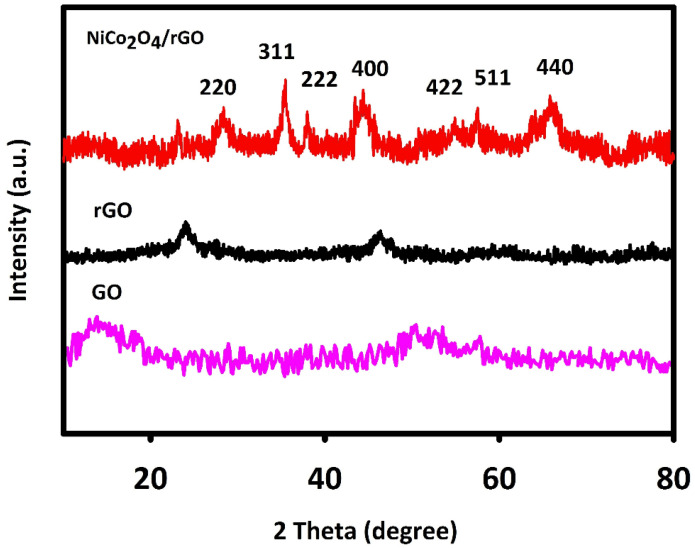
X-ray diffraction (XRD) spectra recorded for fabricated graphene oxide (GO), reduced GO (RGO) and NiCo_2_O_4_/RGO.

The XRD method was used to evaluate NiCo_2_O_4_/RGO and NiCo_2_O_4_ for crystal structure. Figure 1 shows the 2θ angles of XRD peaks and corresponding planes, in line with the previous findings and JCPDS (20-078).

The 2θ values of 64.86°, 59.16°, 55.28°, 44.54°, 38.48°, 36.61° and 31.09° were related to the cubic NiCo_2_O_4_ phase planes of (440), (511), (422), (400), (222), (311) and (220) in line with JCPDS [20-0781] and previous findings. The wide peak (2θ = 25°) corresponded to RGO. GO has a relatively wide characteristic peak at 2θ about 10°, which indicates the graphite was fully oxidized into GO. The mean crystallite size of NiCo_2_O_4_/RGO was calculated to be 70 nm in accordance with the Debye–Scherrer equation.

**Figure 2:**
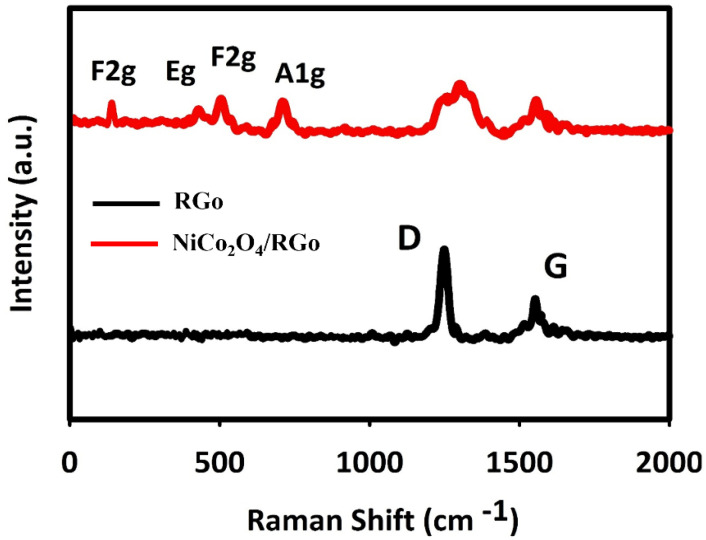
Raman spectra recorded for NiCo_2_O_4_/RGO and RGO.

The Raman spectra recorded for fabricated materials are shown in Figure 2. The wide peaks at 1583 and 1330 cm^−1^ were related to NiCo_2_O_4_/RGO and RGO, corresponding to G and D bands, respectively. The peak of Raman G band was related to in-plane movement of sp^2^ carbons, and the Raman D band peak to sp^3^ (out-of-plane vibrations).

The D/G ratio is higher in the composite RGO than in the pure RGO. The elevated D/G ratio means the RGO hybridization with NiCo_2_O_4_, enhancing the defect density in RGO and defect in carbon layers. The peaks of Ni–O and Co–O vibrations at 654, 501, 456 and 181 cm^−1^ were, respectively, related to A1g, F2g, Eg and F2g of phonon types of NiCo_2_O_4_.

**Figure 3:**
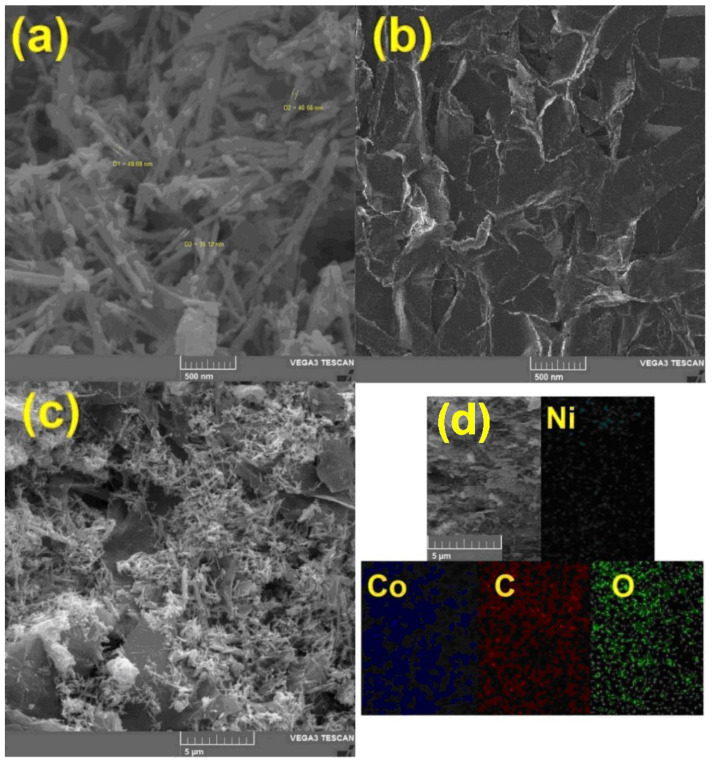
The scanning electron microscopy (SEM) images taken from (**a**) NiCo_2_O_4_ nanorod, (**b**) RGO nanosheets, (**c**) NiCo_2_O_4_/RGO and (**d**) The energy-dispersive X-ray analysis (EDX) mapping of NiCo_2_O_4_/RGO.

To study the surface morphology and also to prove the presence of hybrid constituent elements, scanning electron microscope (SEM) images and energy-dispersive X-ray analysis (EDX) mapping were prepared. Figure 3a shows NiCo_2_O_4_ nanoparticles. The average size of these nanorods is about 40 nm, and in this figure, a uniform rod-shaped morphology can be seen. RGO nanosheets are also shown in Figure 3b. The plate morphology of these two-dimensional materials is also clearly shown in this image. In the case of hybrids used in electrochemical processes, one of the most important parameters is the uniform dispersion of nanomaterials on a substrate. Figure 3c clearly shows the uniform distribution of NiCo_2_O_4_ nanorods on the surface of RGO. The presence of nano-hybrid constituents, including nickel, cobalt, oxygen, and carbon was confirmed by the EDX mapping analysis (Figure 3d).

### 3.2. Electrochemical Responses of Hydroxylamine on the Surface of NiCo_2_O_4_/Reduced Graphene Oxide (RGO)/Screen-Printed Electrode (SPE)

The solution pH values influence the electrochemical responses of HA, highlighting the necessity for optimizing the solution pH to determine the electrocatalytic HA oxidation, which was evaluated in 0.1 M PBS at various pH values (2.0 to 9.0) on the NiCo_2_O_4_/RGO/SPE surface using cyclic voltammetry. The results suggested neutral pH value to achieve the best outcomes of HA electrooxidation on the NiCo_2_O_4_/RGO/SPE surface. Hence, the optimal pH value was selected to be 7.0 for this purpose in the next testing.

**Figure 4:**
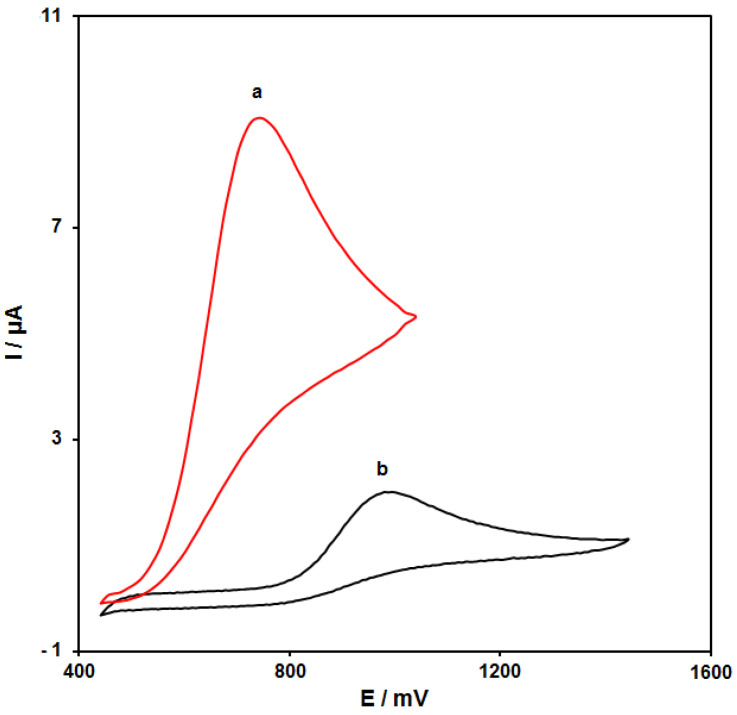
Cyclic voltammograms (CVs) of bare screen-printed electrode (SPE) (a) and NiCo_2_O_4_/RGO/SPE (b) in the presence of 0.1 M phosphate-buffered saline (PBS) at the pH value of 7.0 for detection of HA (100.0 μM) at the scan rate of 50 mV/s.

Figure 4 (curves a and b) shows the cyclic voltammograms (CVs) recorded for electrooxidation of HA (100.0 μM) on the surfaces of bare SPE and NiCo_2_O_4_/RGO/SPE. The findings from the CVs confirmed the best HA oxidation on the NiCo_2_O_4_/RGO/SPE surface at 750 mV, about 250 mV more negative than that on the bare SPE, underlining a significant improvement of HA oxidation signal via the NiCo_2_O_4_/RGO nanocomposite.

### 3.3. Results of Scan Rate Impact

**Figure 5:**
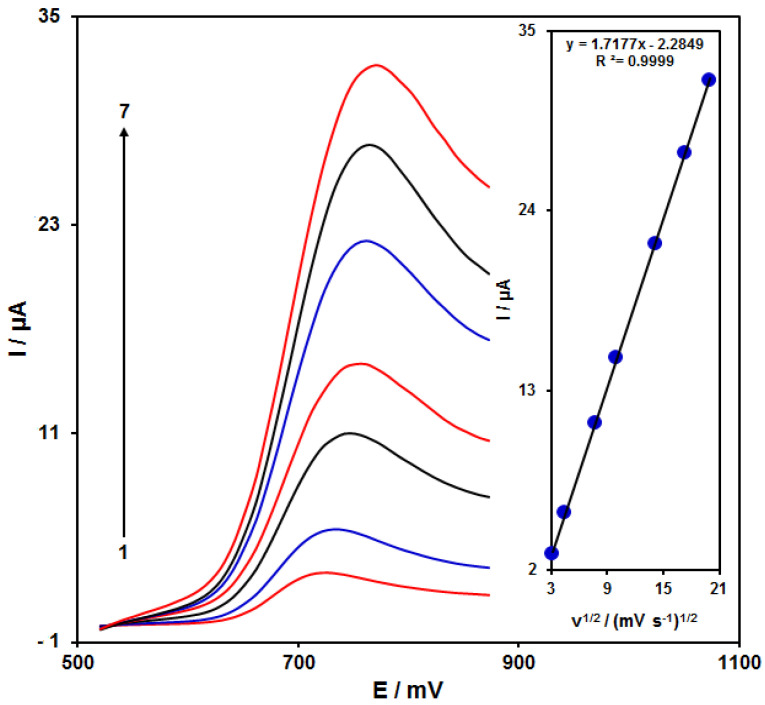
LSVs of NiCo_2_O_4_/RGO/SPE in the presence of 0.1 M PBS at the pH value of 7.0 for detection of HA (150.0 μM) at different scan rates, indicating numbers 1–7 as 10, 20, 60, 100, 200, 300 and 400 mV/s. Inset: anodic peak current variation versus ν^1/2^.

Figure 5 shows the scan rate impact on the HA oxidation current, the results of which indicated an increase in the peak current with increasing scan rate. The oxidation process followed the diffusion-limited reactions obtained from the linear dependence of the anodic peak current (Ip) on the square root of the scan rate (ν^1/2^, 10–400 mV/s).

### 3.4. Chronoamperometric Measurements

Chronoamperometry was employed to evaluate the catalytic HA oxidation on the modified electrode surface in the presence of different HA concentrations on the working electrode set at the potential value of 800 mV. The HA diffusion coefficient was also determined. According to previous findings, the electrochemical current of HA under the mass transport-limited condition could be calculated using the Cottrell method:

I = nFAD^1/2^ C_b_π^−1/2^ t^−1/2^

**Figure 6:**
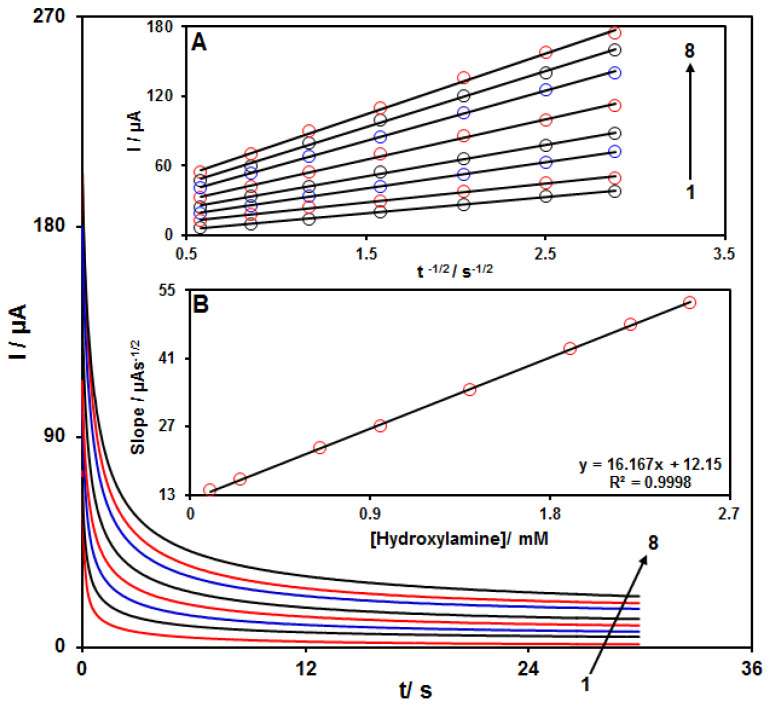
Chronoamperograms for NiCo_2_O_4_/RGO/SPE in the presence of 0.1 M PBS at the pH value of 7.0 for detection of HA at different concentrations, indicating numbers 1−8 as 0.1, 0.25, 0.65, 0.95, 1.4, 1.9, 2.2 and 2.5 mM of HA. Insets: (**A**) Cottrell plot for chronoamperogram findings, (**B**) slope of the plot of straight lines versus HA content.

In this equation, D and C_b_ stand for diffusion coefficient (cm^2^/s) and bulk concentration (mol/cm^3^), respectively. Figure 6A shows the plot of I versus t^−1/2^ on the basis of experiments for various HA specimens. Figure 6B displays the slope of straight line versus HA content. The D value for HA was calculated to be 2.2 × 10^−5^ cm^2^/s based on the Cottrell equation and the slopes obtained.

### 3.5. Calibration Curve and Limit of Detection

**Figure 7:**
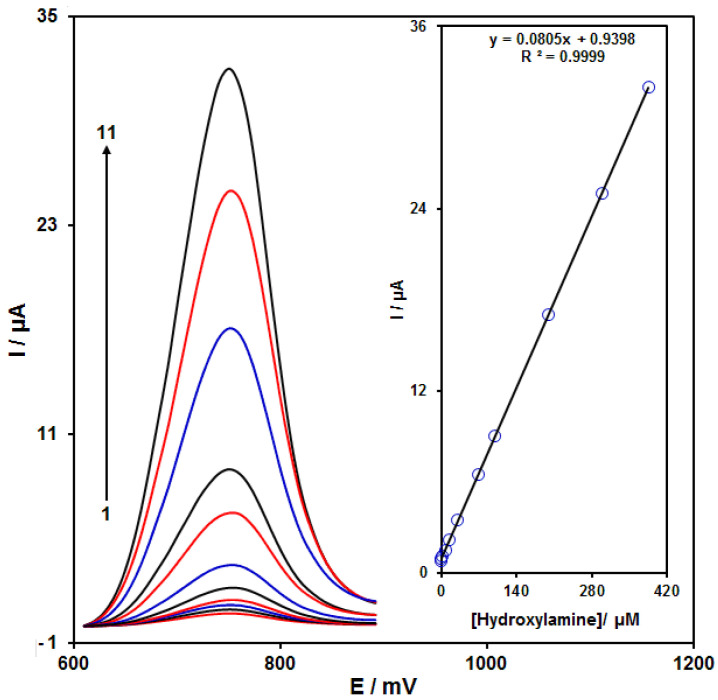
DPVs of NiCo_2_O_4_/RGO/SPE in the presence of 0.1 M PBS at the pH value of 7.0 for detection of hydroxylamine (HA) at different concentrations, indicating numbers 1–11 as 0.007, 0.1, 1.0, 7.5, 15.0, 30.0, 70.0, 100.0, 200.0, 300.0 and 385.0 µM of HA. Inset: plot of peak current as a function of different HA concentrations (0.007–385.0 µM).

The peak currents of HA electro-oxidation on the NiCo_2_O_4_/RGO/SPE surface were used for the HA detection. Because hypersensitivity and appropriate analytical features are the advantages of differential pulse voltammetry (DPV), different HA concentrations in the NiCo_2_O_4_/RGO/SPE and PBS (0.1 M) were used for DPV analysis as shown in Figure 7 (response time = 6 s). The peak currents of HA oxidation on the surface of NiCo_2_O_4_/RGO/SPE depends linearly on HA concentrations (0.007 to 385.0 μM). The linear equation was as y = 0.0805 x + 0.9398, the correlation coefficient was estimated at 0.9999, but the limit of detection (3σ) was estimated at 2.0 nM. The LOD and linear range of HA at the NiCo_2_O_4_/RGO/SPE electrode presented in this work were compared with the reported modified electrodes and were given in Table 1.

### 3.6. Interference Study

The impact of numerous interference substances on the detection of HA was investigated. The tolerance limit was taken as the maximum of the foreign materials concentration that resulted in about ±5% relative error in the detection. The results indicated that glucose, lactose, sucrose, urea, sulfite, hydrazine, phenol, thiosulphate, bisphenol A, Mg^2+^, Ni^2+^, K^+^, Li^+^, Mn^2+^, Cr^2+^, Zn^2+^, CN-, Br^−^, and SCN^−^ did not show interference in the determination of HA.

### 3.7. Real Sample Analysis

The fabricated NiCo_2_O_4_/RGO/SPE was used for the detection of HA present in varied water specimens using the method of standard additions. The HA concentration and recovery rate are shown in Table 2. An excellent recovery rate was found for the HA, and mean relative standard deviation (RSD%) confirmed the reproducibility. The applicability of NiCo_2_O_4_/RGO/SPE sensor was confirmed by sensitive detection of HA concentrations in drinking, tap and river water specimens.

## 4. Conclusions

In this work, we prepared NiCo_2_O_4_/RGO nanocomposite by a simple method. The successful preparation of this nanocomposite was revealed by FESEM, EDX mapping, XRD, and Raman spectroscopy. Then, we developed a novel voltammetric hydroxylamine sensor through the surface modification of SPE with NiCo_2_O_4_/RGO nanocomposite as a signal amplifier for HA detection. The oxidation peak currents of HA presented a good linear relationship with the concentrations in the range from 0.007 to 385.0 μM with a detection limit of 2.0 nM. Moreover, the detection of HA in water samples by the proposed sensor showed satisfactory results.

## Figures and Tables

**Table 1:** Comparison of the efficiency of the NiCo_2_O_4_/RGO/SPE electrode with literature modified electrodes for HA determination.

Electrochemical Sensor	Electrochemical Method	Linear Range	Limit of Detection	Ref.
Fullerene-functionalized carbon nanotubes/ionic liquid nanocomposite/glassy carbon electrode	DPV	1.0–300.0 μM	28 ± 2 nM	[10]
Graphene oxidde/TiO_2_/SPE	DPV	0.1–300 μM	0.065 µM	[11]
Gold nanoparticles/cetyltrimethyl ammonium bromide/graphene oxide/glassy carbon electrode	Amperometry	10–1000 μM	3.5 μM	[12]
Prussian blue-multi-walled carbon nanotubes/glassy carbon electrode	Amperometry	1.5 µM–2.0 mM	-	[13]
Gold nanoparticles—polypyrrole nanowire/glassy carbon electrode	DPV	1–500 μM	0.21 μM	[14]
1-benzyl-4-ferrocenyl-1H-[1,2,3]-triazole/carbon nanotube/glassy carbon electrode	Square wave voltammetry	4.0 × 10^−7^– 6.75 × 10^−4^ M	28.0 ± 1.0 nM	[15]
NiCo_2_O_4_/RGO/SPE	DPV	0.007–385.0 μM	2.0 nM	This Work

**Table 2:** Recoveries for detection of hydroxylamine in water specimens (*n* = 5).

Sample	Spiked	Found	Recovery (%)	R.S.D. (%)
Drinking water	0	-	-	-
	5.0	4.9	98.0	3.2
	7.0	7.3	104.3	2.7
	9.0	9.1	101.1	1.9
	11.0	10.9	99.1	2.4
Tap water	0	-	-	-
	4.5	4.6	102.2	2.9
	6.5	6.3	97.0	3.5
	8.5	8.4	98.8	1.6
	10.5	10.6	100.9	2.5
River water	0	-	-	-
	5.5	5.4	98.2	2.7
	7.5	7.8	104.0	2.1
	9.5	9.6	101.0	3.6
	11.5	11.4	99.1	2.0

## Data Availability

The data presented in this study are available on request from the corresponding author.
